# Principles and Practice of Surgery

**Published:** 1872-11-01

**Authors:** E. Andrews


Principles and Practice of Surgery. By Frank Hastings Hamilton, A.M., M.D., LLD., etc., etc. Published by William H. Wood & Co., New York. 1872.

Prof. Hamilton first became known, nationally, by means of his statistics upon fractures, by means of which he demonstrated the necessary imperfection of results in most cases of broken bones. This work, by showing the absurdity of demanding perfect results in the treatment of fractures, furnished a weapon of defence in mal-practice suits, which was made use of with excellent effect, and did much to put an end to the mania for prosecuting physicians, which was then going like an epidemic over the country.

Afterwards, Prof. H. published a work on fractures and dislocations, which, though carelessly written in certain parts, was full of thorough investigation in others, and is probably the best book in the world on that subject. He also issues an excellent book on military surgery, which is widely known.

The present work, therefore, is sure to command attention. It is an octavo in one volume, of 950 pages, and illustrated with 467 wood cuts. Part first is on general surgery. It comprises chapters on inflammation, abscess, ulceration, gangrene, tetanus, wounds, venereal diseases, aneurism, etc., embracing all affections and operations which are not necessarily limited to a particular region. Part second is on regismal surgery. In the chapters on syphilis, Dr. Hamilton seems to lean to the ideas of the dualists, but does not very clearly enunciate any opinion on the subject.

In respect to antispeptic surgery, the book is extremely vague and unsatisfactory, which, considering the present state of knowledge on that subject, is to be regretted.

In fractures and dislocations, on the contrary, the discussion is clear, full, and admirable. In dislocations of the hip-joint, the author has availed himself of Bigelow's investigations, and is the first author of a general surgery, to put that topic on its new and revolutionized basis. On the whole, this is one of the best text-books upon surgery which we have ever seen, and we recommend it highly to the profession.

E. Andrews, M. D.

Transactions of the Twenty-second Anniversary meeting of the Illinois State Medical Society, held at Rock Island, May 21st and 22d, 1872.

A volume of 230 pages, containing the usual assortment of valuable and interesting reports and essays.

